# Advillin Is Expressed in All Adult Neural Crest-Derived Neurons

**DOI:** 10.1523/ENEURO.0077-18.2018

**Published:** 2018-09-13

**Authors:** Diana V. Hunter, Brittney D. Smaila, Douglas M. Lopes, Jun Takatoh, Franziska Denk, Matt S. Ramer

**Affiliations:** 1 International Collaboration on Repair Discoveries (ICORD), the University of British Columbia, Vancouver, British Columbia V5Z1M9, Canada; 2 Wolfson Centre for Age-Related Diseases, King’s College London, London SE1 1UL, United Kingdom; 3 Department of Neurobiology, Duke University School of Medicine, Durham, NC 27710

**Keywords:** advillin, autonomic neurons, enteric neurons, parasympathetic neurons, sensory neurons, sympathetic neurons

## Abstract

Promoter-based genetic recombination (via, e.g., Cre-lox) is most useful when all cells of interest express a particular gene. The discovery that the actin-binding protein advillin is expressed in all somatic sensory neurons has been exploited repeatedly to drive DNA recombination therein, yet specificity of expression has not been well demonstrated. Here, we characterize advillin expression amongst sensory neurons and in several other neural and non-neural tissues. We first validate an advillin antibody against advillin knock-out tissue, advillin promoter-driven EGFP, and advillin mRNA expression. In the dorsal root ganglion (DRG), advillin is enriched in non-peptidergic nociceptors. We also show that advillin expression, and advillin promotor-driven EGFP and Cre-recombinase expression, occurs in multiple tissues including the dorsal habenula of the epithalamus, endocrine cells of the gut, Merkel cells in the skin, and most strikingly, throughout the autonomic nervous system (sympathetic, parasympathetic, and enteric neurons) in mice, rats, and non-human primates. In the mouse pelvic ganglion, advillin immunoreactivity is most intense in pairs of small neurons, and concentrated in spine-like structures on the axon initial segment contacted by sympathetic preganglionic axons. In autonomic targets (iris and blood vessels), advillin is distributed along cholinergic parasympathetic axons and in sympathetic varicosities. Developmentally, advillin expression is absent from sympathetics at postnatal day 4 but begins to emerge by day 7, accounting for previous reports (based on embryonic expression) of advillin’s specificity to sensory neurons. These results indicate that caution is warranted in interpreting previous studies in which advillin-driven genomic editing is either constitutive or performed after postnatal day 4.

## Significance Statement

Cell-specific promoters are useful for effecting DNA recombination (e.g., via Cre-lox) for studies of lineage and gene function. Accordingly, advillin, an actin-binding protein expressed in all somatic sensory neurons, has proven valuable in manipulating gene expression therein. Whether advillin expression is restricted to sensory neurons, which is crucial to ascribing primary afferent-specific roles of particular genes, has not been established. Here, we show that advillin is also expressed throughout the adult autonomic and enteric nervous systems, the habenula, and endocrine cells of the skin and gut. Thus, while advillin promoter activity remains useful in achieving recombination in sensory neurons (and some other cells), caution is warranted in interpreting results of experiments in which outcomes depend on multiple advillin-expressing tissues.

## Introduction

The ability to manipulate gene expression within specific cells using recombinase driver (e.g., Cre, Dre, Flp) mice has proved a useful tool in studying gene function. Identifying appropriate promoters, expressed exclusively and ubiquitously within a cell population of interest, to drive recombinase expression is a crucial and challenging first step. This is particularly true for primary sensory neurons of the dorsal root ganglion (DRG), which represent an extremely heterogeneous population that have significant overlap in gene expression with other peripheral neurons (Usoskin et al., 2015). Multiple lines have been created that target subpopulations of sensory neurons based on the expression of sensory receptors, channels or neuropeptides. In the DRG, for example, Na_v_1.8-Cre-recombinase expression occurs predominantly in nociceptive but not mechanoreceptive neurons allowing for manipulation of genes within this subset of nociceptors (Stirling et al., 2005). Other sensory neuron populations can be similarly targeted with other subtype-specific promoters (e.g., parvalbumin for proprioceptors, Ret for low-threshold cutaneous mechanoreceptors; Niu et al., 2013; Chiu et al., 2014). However, these promoters target only some DRG neurons and their expression in other tissues reduces their overall utility. As such, the discovery of the expression patterns of the advillin gene generated significant interest.

Advillin is a member of a gelsolin/villin superfamily of actin binding and regulatory proteins (Marks et al., 1998). Identified through a screen of an adult murine brain cDNA, advillin was found to have a high degree of homology with villin, gelsolin and adseverin, all of which are important for control of actin organization (Marks et al., 1998; Silacci et al., 2004). Functional experiments suggest that advillin is important for neurite growth and for the development of neuronal cells which form ganglia (Marks et al., 1998; Ravenall et al., 2002). Its strong expression in DRG and trigeminal ganglion neurons at embryonic and adult stages led to the production of multiple mouse lines that used the advillin expression for the purposes of labeling, or manipulating genes, within all primary sensory neurons.

Initially, a knock-in mouse with the start codon of advillin replaced by the axonal tracer human placenta alkaline phophastase was created for the characterization of advillin expression and visualization of sensory endings and central terminal arborizations (Hasegawa et al., 2007). The results suggested that advillin was expressed almost exclusively in sensory neurons. This lead to the development of a mouse with Cre-recombinase under the regulatory elements of the advillin gene for the excision of floxed genes within neurons of the peripheral ganglia (Zurborg et al., 2011). Subsequently, Lau et al. (2011) recombineered the knock-out mouse with a tamoxifen-inducible CreERT2 recombinase that they also reported was only expressed in sensory neurons on tamoxifen dosing.

Since sensory and sympathetic axons are intermingled in target tissues, peripheral nerves, and sympathetic ganglia (Edgar, 2007; Wang et al., 2014), and communication between these systems is important in conditions such as pain and inflammation (Raja, 1995; Pongratz and Straub, 2014), we sought to take advantage of advillin-based reporters and Cre drivers to study the specific role(s) of sensory neurons in these conditions. However, we immediately found that advillin is expressed throughout the peripheral autonomic nervous system. This was surprising, given assertions to the contrary, but partially supported by limited descriptions of expression patterns of advillin in the earlier literature (Marks et al., 1998; Ravenall et al., 2002; Zurborg et al., 2011). This led us to undertake a more thorough investigation of the expression of advillin in the entire peripheral nervous system and peripheral target tissue of the adult mouse. Along the way we characterize advillin’s differential expression in the mouse DRG, describe novel advillin-positive axonal spines in pelvic ganglion neurons (mixed sympathetic/parasympathetic), and confirm advillin expression throughout the entire peripheral autonomic nervous system of mice and selected autonomic ganglia in rats and monkeys. Finally, we show that the likely source of confusion regarding advillin’s specificity is rooted in development: advillin only begins to be expressed in sympathetic and enteric neurons postnatally.

## Materials and Methods

### Animals and tissues

Observations were made on three P17 advillin knock-out mice and two wild-type littermates (a generous gift of Prof. Fan Wang, Duke University), five wild-type adult male C57Bl6 mice, two STOCK Tg (Avil-EGFP) QD84Gsat/Mmucd bred in-house for several generations on a CD1 background (“advillin-EGFP,” a BAC transgenic), three male advillin-CreERT2 reporter mice [B6.Cg-Tg(Avil-icre/ERT2)AJwo/J crossed with B6.Cg-*Gt(ROSA)26Sor^tm14(CAG-tdTomato)Hze^*/J from The Jackson Laboratory (also a BAC transgenic), killed 4 d after 3 d of 75 mg/kg tamoxifen treatment], five adult male Wistar rats, and two rhesus macaques (DRG and sympathetic ganglion tissue a gift of Qingan Zhu, Department of Spine Surgery, Nanfang Hospital, The Southern Medical University, Guangdong, China). We also used P4 (*n* = 6), P7 (*n* = 6), and P10 (*n* = 4) advillin-EGFP and wild-type littermate pups. Adult animals were transcardially perfused with 4% formaldehyde in 0.1 M phosphate buffer (PB). Pups were immersion-fixed for one week after their abdomens, thoraxes, and skulls were opened. From all adult animals, we removed dorsal root ganglia and sympathetic chain ganglia (superior cervical, stellate, and upper thoracic). From all adult rodents we also removed otic ganglia (Al-Hadithi and Mitchell, 1987; cranial parasympathetic ganglia responsible for parotid salivary activity) and major pelvic ganglia (mixed sympathetic and parasympathetic). From adult wild-type and advillin-EGFP mice we harvested adrenal glands, and hairy and glabrous skin of the hindpaw. From rats we also removed irises. We examined brains (specifically habenulae) and spinal cords from adult wild-type mice. We removed superior cervical ganglia from half of the P7 and P10 mouse pups. The other half we sectioned the entire skinned carcass en bloc from the external auditory meatus to mid-cervical region. From all pups we harvested segments of small intestine.

### Tissue processing and immunohistochemistry

Tissues were either processed as whole mounts (sympathetic and parasympathetic ganglia, and duodenum from advillin-EGFP mice, duodenum and major pelvic ganglia from wild-type mice, and rat iris), or cut at 20 (adult) or 30 (en bloc pup carcasses) microns on a cryostat and thaw-mounted onto glass slides (all other tissues as well as duodenum, except brain and spinal cord), or cut at 50 μm on a cryostat and processed for free-floating section immunohistochemistry (brain and spinal cord).

Material was first incubated in 10% normal donkey serum in PBS plus 0.2% Triton X-100, plus 0.02% sodium azide for 20 min (sections) for 2 h (whole mounts). Primary antibodies used (over one night for sections, over two nights for whole mounts) were rabbit anti-advillin (1:500, abcam 72210), mouse anti-βIII-tubulin (1:500, Sigma, T8660), chicken anti-MAP2 (1:5000, abcam, ab5392), goat anti-choline acetyltransferase (ChAT, 1:200, Millipore, AB144P), sheep anti-tyrosine hydroxylase (TH; 1:100, Pel-Freez Biologicals, P60101), and mouse anti-dopamine β-hydroxylase (DβH, 1:500, Millipore, MAB308). Secondary antibodies were applied at 1:500 (2 h for sections and overnight for whole mounts), and conjugated to Cy3 (donkey anti-rabbit, Jackson ImmunoResearch, 711-165-152), Alexa Fluor 650 (Donkey anti-goat, abcam, ab96934), or Alexa Fluor 488 (donkey anti-mouse, Jackson ImmunoResearch, 715-545-151; donkey anti-sheep, Invitrogen, A11015; donkey anti-chicken, Sigma, SAB4600031). In some cases, Alexa Fluor 488-conjugated lectin GS-IB4 (Invitrogen, 121411, 1 mg/ml) was added to the primary antibody mixture at a 1:500 dilution. Between primary and secondary antibody exposure, tissue was washed in PBS (3 × 10 min), and following secondary antibody incubation slides were washed again and coverslipped in either ProLong Gold anti-fade reagent with DAPI (Invitrogen), or Vectashield hard-mount with DAPI (Vector Laboratories).

### Imaging and analysis

Laser scanning confocal microscopy was conducted using a Zeiss LSM 800 mounted on a Zeiss Image Z.2 microscope. Images were captured using Zen (Blue) software, and images were built using Adobe Photoshop CC 2014.

We used ImageJ (FIJI) to measure relative intensity of EGFP fluorescence, advillin immunoreactivity and IB4-488 intensity in 8-bit dually stained images of L4 and L5 DRG sections (either advillin and EGFP or advillin and IB4-488) along with neuronal profile area in DRG neurons. In comparing advillin and EGFP intensities, we captured a single image from each of the L4 and L5 ganglia from two advillin-EGFP mice. For IB4-488 staining, we captured a single image from each of the L4 and L5 DRGs from four wild-type mice. For advillin-EGFP mice, all DRG neuronal profiles were individually traced from the EGFP channel. In advillin/IB4 colocalization studies, the advillin channel served as the reference. Each section yielded 100–150 profiles.

Scatterplots were prepared in Sigmaplot 2001 (SPSS), and linear regression analysis was conducted using Prism (GraphPad).

## Results

### Validation of an advillin antibody

**Figure 1.**
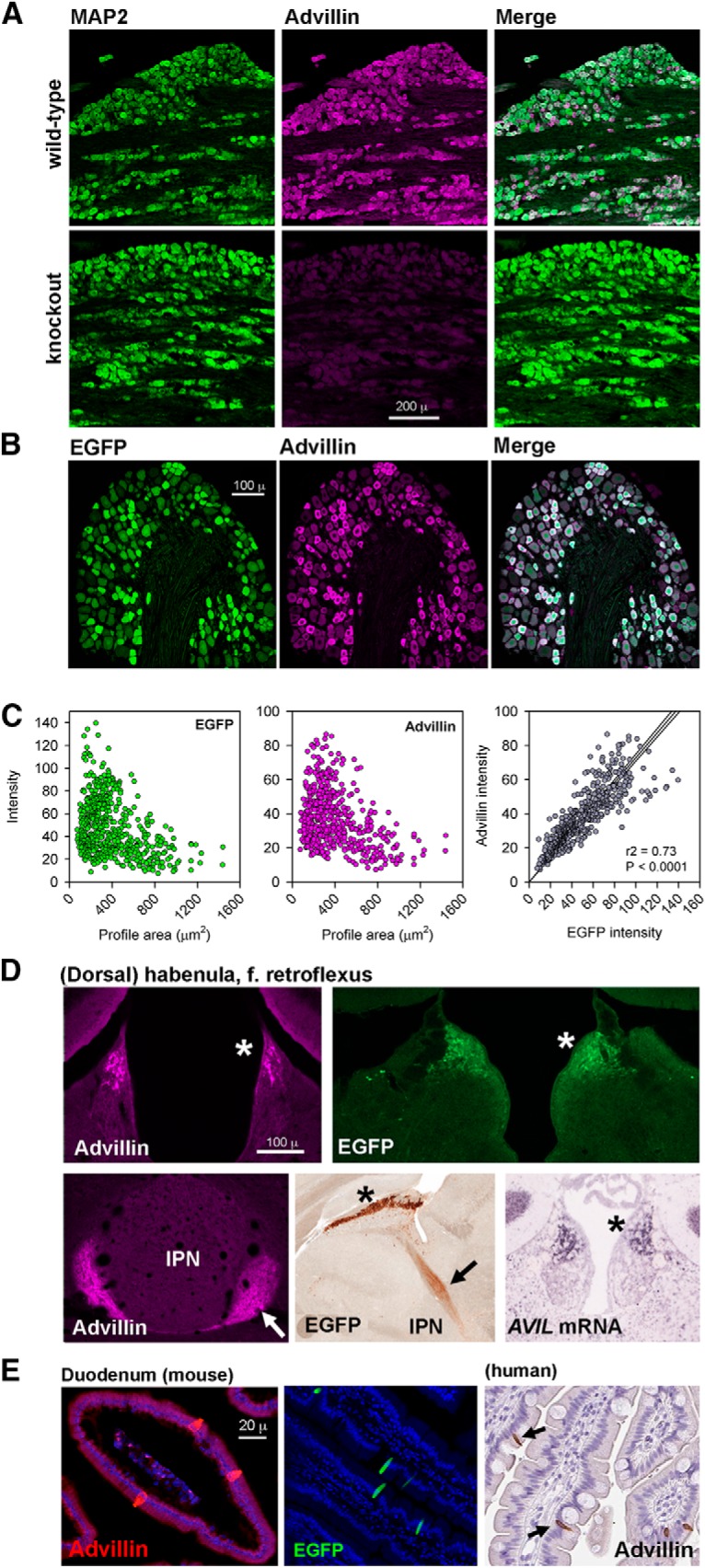
Validation of an advillin antibody (abcam ab72210). ***A***, MAP2 and advillin expression in wild-type and advillin knock-out mice. ***B***, DRG from advillin-EGFP mouse labeled with anti-advillin antibody. ***C***, Scatterplots of EGFP or advillin intensity versus neuronal profile diameter (left, middle) and quantification of the tight correlation between EGFP and advillin intensity. ***D***, Advillin immunoreactivity in a wild-type mouse, *AVIL* mRNA in a wild-type mouse, and EGFP immunoreactivity in the advillin-EGFP mouse in the dorsal habenula (asterisks) and the fasciculus retroflexus (arrows; IPN, interpeduncular nucleus of the midbrain). ***E***, Advillin immunoreactivity (wild-type mouse) and EGFP fluorescence (advillin-EGFP mouse) in endocrine cells of the duodenum. Inset shows advillin immunoreactivity in the human duodenum with a different antibody. Image credits: bottom middle in ***D***, GENSAT (http://www.gensat.org/imagenavigator.jsp?imageID=86610); bottom right in ***D***, Allen Institute (http://mouse.brain-map.org/experiment/show?id=72340234); right panel of ***E***, Human Protein Atlas (https://www.proteinatlas.org/ENSG00000135407-AVIL/antibody#cbox).

The advillin antibody did not label trigeminal sensory neurons from advillin knock-out mice (Fig. 1*A*), but did in wild-type littermates. We examined the relationship between EGFP and advillin expression in the DRG of advillin-EGFP mice. All profiles were both EGFP and advillin-positive, and there was no advillin staining in any EGFP-negative cells (Fig. 1*B*). Smaller cells had particularly high EGFP fluorescence and advillin immunoreactivity, and there was a strong and significant correlation between EGFP and advillin intensity (Fig. 1*C*).

We searched online resources (www.alleninstitute.org; www.gensat.org; www.proteinatlas.org) for advillin expression in other tissues. Advillin promoter activity (driving EGFP), and advillin mRNA is expressed in the brain not only in mesencephalic trigeminal nucleus sensory neurons (not shown), but also in the dorsal habenula (Fig. 1*D*). In advillin-EGFP mice, EGFP is also in axons of dorsal habenular neurons making up the fasciculus retroflexus projecting to the ventral midbrain. Advillin immunohistochemistry matched both advillin mRNA and EGFP distribution (Fig. 1).

Advillin protein (as determined using a rabbit polyclonal antibody directed against an 86-aa protein fragment; Atlas Antibodies catalog #HPA058864, RRID:AB_2683838) is also expressed at high levels in endocrine cells of the human duodenum (www.proteinatlas.org). We found intense advillin immunoreactivity in wild-type mouse duodenal endocrine cells and intense EGFP fluorescence in the same cells in the advillin-EGFP duodenum (Fig. 1*E*).

**Figure 2.**
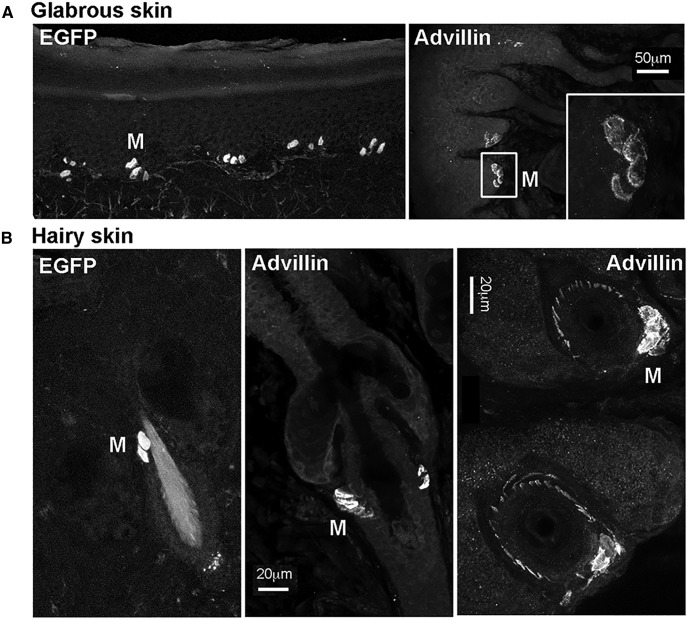
Advillin expression by Merkel cells (M). ***A***, Glabrous skin. ***B***, Hairy skin. Leftmost panels show advillin promoter-driven EGFP fluorescence. Other panels show advillin immunoreactivity in wild-type mice. The bottom right panel additionally shows hair follicles cut parallel to the skin surface, surrounded by advillin-positive axons.

In the skin, individual advillin-positive axons were occasionally apparent around hair follicles (Fig. 2), but in general, they were undetectable outside of fasciculated nerves (see also below). Merkel cells, however, both at the dermal-epidermal border and around and hair follicles, were evident by both EGFP fluorescence in advillin-EGFP mice, and by advillin immunohistochemistry in wild-type mice (Fig. 2). This is in agreement with a previous report of advillin expression by Merkel cells (Ranade et al., 2014).

### Advillin is expressed at high levels in IB4-binding DRG neurons

**Figure 3.**
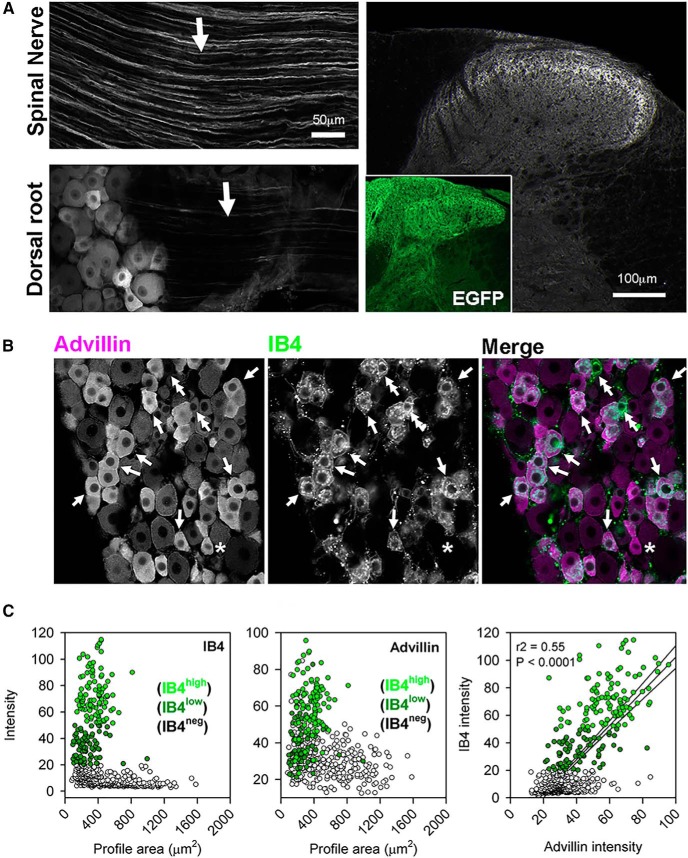
Advillin is enriched in nonpeptidergic nociceptors. ***A***, Advillin immunoreactivity is strong in the spinal nerve but weak in the dorsal root. In the cord, intense advillin immunoreactivity is only apparent in inner lamina II. Inset, Advillin promoter-driven EGFP is distributed to all primary afferent axons. ***B***, Advillin immunoreactivity is enriched in IB4-binding neurons (arrows). Double arrows indicate rare IB4-binding neurons with weak advillin immunoreactivity, and the asterisk indicates an even more rare IB4-negative neuron with positive advillin immuoreactivity. ***C***, Scatterplots showing IB4 binding and advillin intensity (intensity of IB4 binding indicated by brightness of green, IB4-negative profiles indicated by unfilled circles) as a function of profile area. There is a strong, significant correlation between IB4-binding and advillin immunoreactivity.

Advillin immunoreactivity was intense in the peripheral processes of DRG neurons in the spinal nerve, but weak or absent in the dorsal root (Fig. 3*A*), indicating preferential transport to the periphery in most axons. Advillin immunoreactivity in the spinal cord was detectable only in sensory terminals innervating inner lamina II (IIi), despite the fact that all terminals can be visualized with EGFP immunohistochemistry in advillin-EGFP mice (Fig. 3*A*, inset). Since non-peptidergic (IB4-binding) nociceptive axons are those which innervate lamina IIi (Snider and McMahon, 1998), we asked whether advillin intensity of DRG neurons was predictive of IB4 binding. Indeed, the vast majority of IB4-positive cells also had high advillin intensity (Fig. 3*C*). The converse was also true, reflected in a strong significant correlation between advillin and IB4-488 intensity.

### Advillin is expressed across species in all classes of autonomic neurons

**Figure 4.**
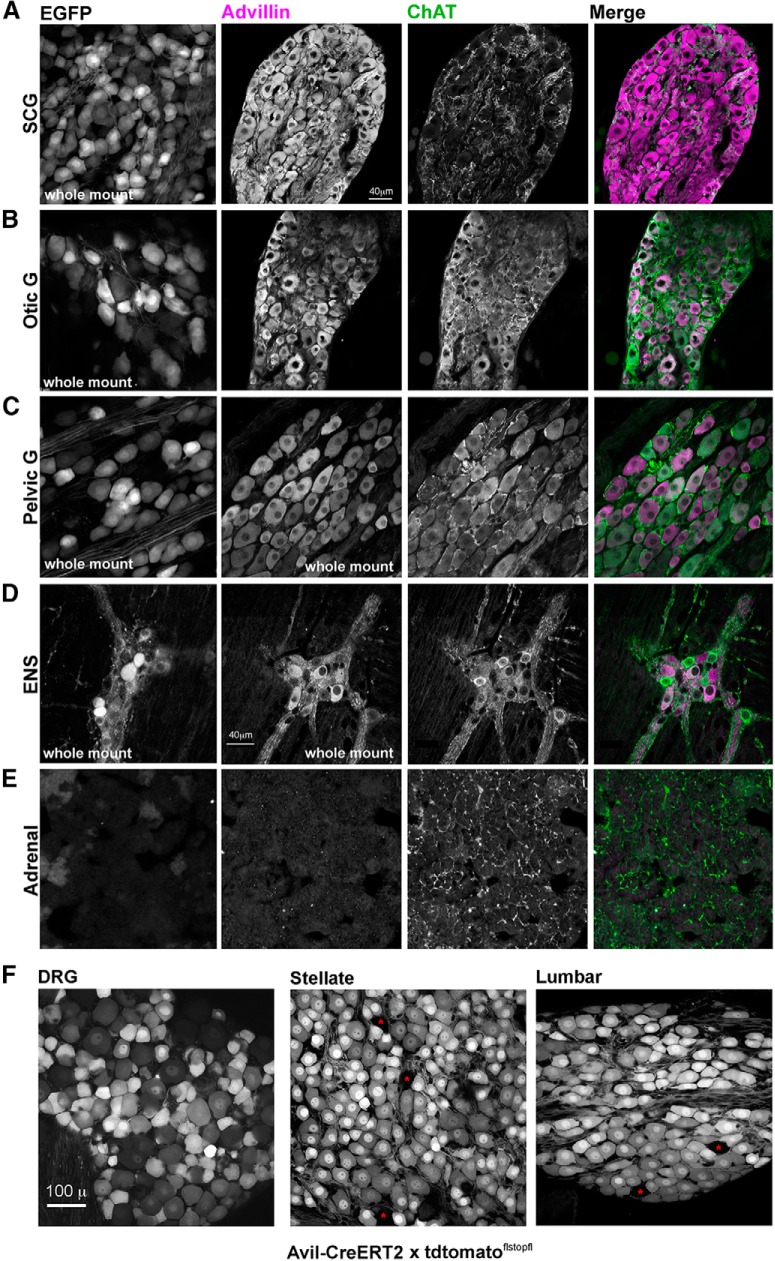
Advillin is expressed throughout the mouse autonomic neuronal lineage. In ***A–E***, the leftmost column is EGFP fluorescence from the advillin-EGFP mouse. ***A***, SCG (sympathetic). ***B***, The otic ganglion (cranial parasympathetic). **C**, major pelvic ganglion (mixed sympathetic parasympathetic). ***D***, Enteric neurons (Meissner’s plexus). ***E***, Advillin is not expressed in the adrenal medulla. ***F***, tdtomato fluorescence in the DRG and stellate and lumbar sympathetic chain ganglia following tamoxifen-induced nuclear translocation of advillin promoter-driven CreERT2 expression in a ^fl^stop^fl^-tdtomato reporter mouse. Red asterisks indicate rare non-recombined neurons.

Figure 4 represents a survey of neural crest-derived neurons in advillin-EGFP mice and wild-type mice processed for advillin immunohistochemistry. Both advillin promoter-driven EGFP and advillin protein are expressed in sympathetic (superior cervical), cranial parasympathetic (otic), mixed sympathetic/parasympathetic (major pelvic ganglion), and enteric neurons throughout the gastrointestinal tract (duodenum shown). In the pelvic ganglion where there are ChAT-positive and ChAT-negative neuronal phenotypes, there was no obvious pattern of (as there was in the DRG) differential advillin staining intensity between populations. Enteric neurons, on the other hand, tended to be large, advillin-rich and ChAT-negative, or smaller, advillin-poor and ChAT-positive. Advillin expression was absent from the adrenal medulla (Fig. 4*E*), despite being of the sympathetic lineage.

Differences in advillin immunoreactivity (or EGFP fluorescence) among autonomic neurons may indicate that some may in fact not express advillin at all. We therefore examined reporter expression in sensory and sympathetic neurons in advillin-CreERT2 mice crossed with floxed-stop ROSA26-tdtomato mice 4 d following 3 d of tamoxifen treatment. In these mice, reporter expression is far less dependent on the level of ROSA26 activity (which ought to be high and ubiquitous), but more on whether the advillin promoter is active at all. In the DRG, reporter expression varied widely across neurons, but inversely with cell size, most likely reflecting consistent reporter transcription but a limited half-life, and widely-varying cytoplasmic volumes (Fig. 4*F*). In the stellate ganglion where neuronal size is intermediate but far more uniform, all but a few (>98%) neurons (which probably had not undergone recombination) were brightly fluorescent (Fig. 4*F*).

**Figure 5.**
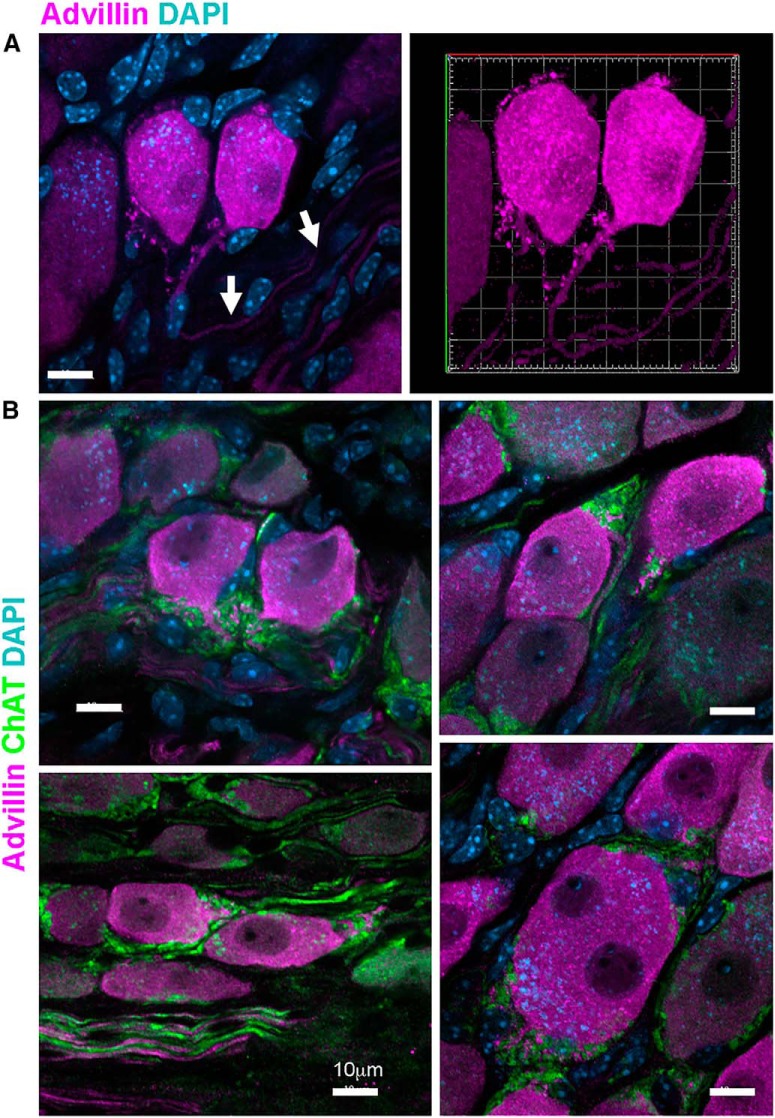
Advillin in spine-like structures on the pelvic ganglion neuron axonal initial segment. ***A***, Confocal stack and 3D reconstruction of an advillin-intense neuronal pair with spine-like structures projecting from the axonal initial segment (arrows). ***B***, Examples of advillin-bright pairs with axonal spines. ChAT-positive preganglionic axons contact neurons at these spines. The lower right panel of ***B*** shows an occasional binucleate neuron, also advillin-intense and with axonal spines.

In mouse pelvic ganglia, intense advillin immunoreactivity defined pairs of small neurons, very similar in size and shape (Fig. 5*A*). Pelvic ganglion neurons typically have no dendrites, but from the axon initial segment of these small bright pairs projected advillin-rich spine-like stuctures to which were apposed ChAT-positive (but advillin-negative) preganglionic axons (Fig. 5*A*). Occasional, large binucleate pelvic ganglion neurons were also intensely-stained for advillin, and had similar axonal spines (Fig. 5*B*).

**Figure 6.**
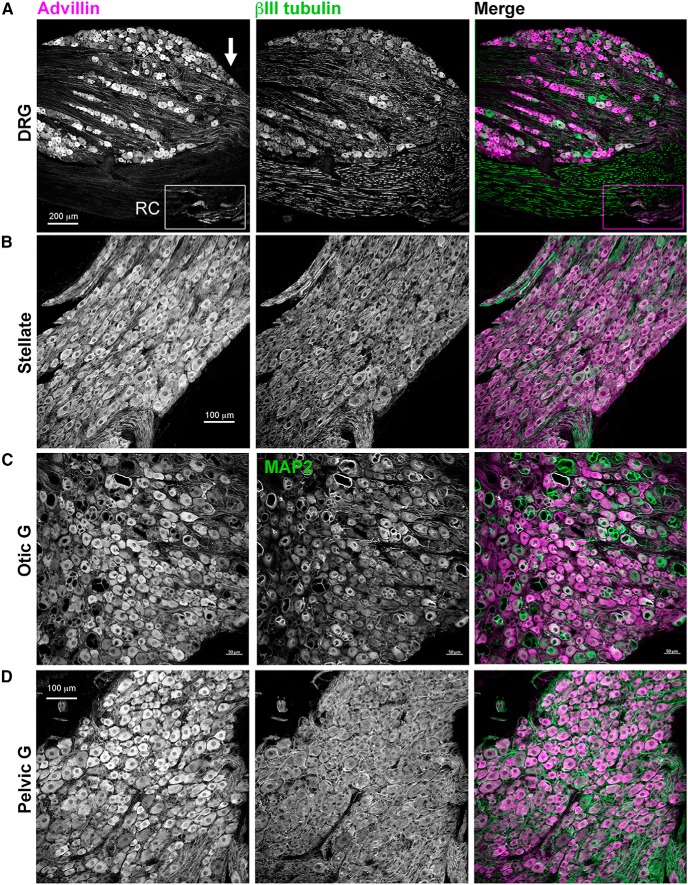
Advillin expression in rat sensory and autonomic neurons. ***A***, DRG. Note intense advillin immunoreactivity at the distal pole of the DRG (arrow), but not in the dorsal root. RC, rami communicantes containing sympathetic axons. ***B***, Stellate ganglion (sympathetic). ***C***, Otic ganglion (cranial parasympathetic). ***D***, Major pelvic ganglion (mixed sympathetic/parasympathetic).

**Figure 7.**
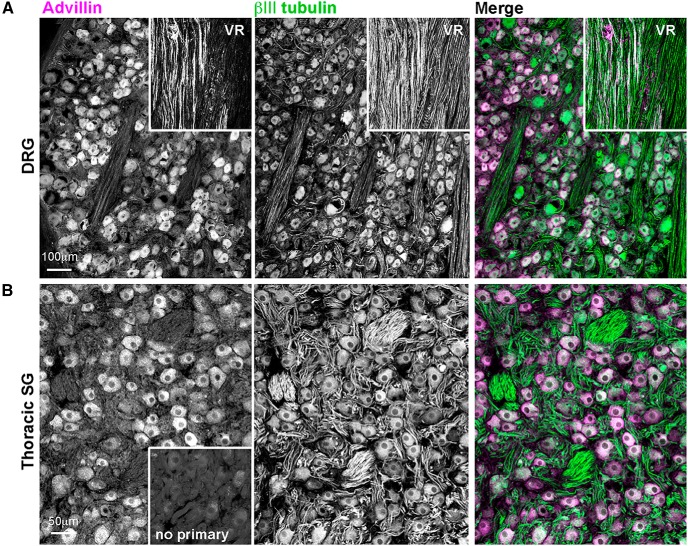
Advillin expression in non-human primate sensory and autonomic neurons. ***A***, DRG. VR, ventral root (advillin-negative). ***B***, Thoracic sympathetic chain ganglion.

Advillin was also expressed in rat DRG, sympathetic, and parasympathetic ganglia (Fig. 6) and in the primate DRG and sympathetic chain (Fig. 7). In both species, we found intense advillin immunoreactivity in the peripheral, but not central, projections of DRG neurons (Fig. 6*A*, arrow), as was the case in mice.

### Advillin immunoreactivity in autonomic target tissues

**Figure 8.**
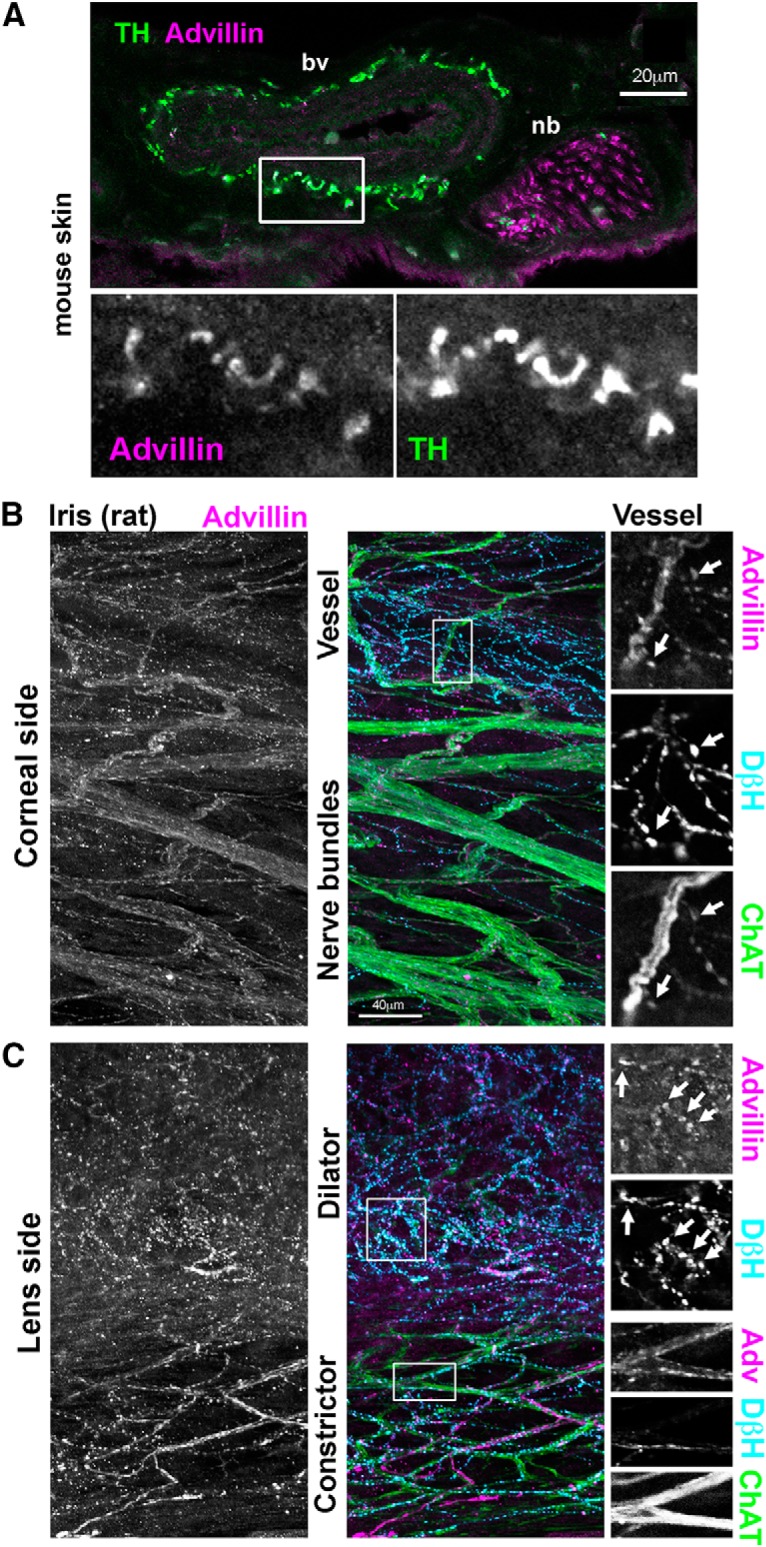
Axonal advillin expression in sympathetic target tissues. ***A***, Mouse skin. Note colocalization between TH and advillin around a blood vessel (bv). A fasciculated nerve bundle (nb) is also apparent. ***B***, ***C***, Whole-mount rat iris. ***B***, Corneal surface of the iris showing heavily-innervated bv and ChAT-positive nbs. ***C***, Lens surface of the iris showing sympathetic and parasympathetic innervation of the dilator and constrictor muscles, respectively.

We next asked whether advillin immunoreactivity could be detected in autonomic target tissues. In mouse skin, advillin labeled fasciculated nerve bundles, as well as TH-positive axons innervating arteries (Fig. 8*A*) and sweat glands (data not shown). Interestingly, advillin immunoreactivity was not detectable in individual (sensory) axons outside of fasciculated nerves. We also examined the rat iris as a target of both sympathetic and parasympathetic innervation. On the corneal surface of the iris, advillin immunoreactivity was present in sympathetic axons innervating blood vessels, where it was concentrated in varicosities, and along cholinergic nerve bundles (Fig. 8*B*). On the lens side, advillin was clearly present in sympathetic axons of the dilator smooth muscle (again, in varicosities), and in cholinergic axons of the constrictor, where it was more uniformly distributed along ChAT-positive axons.

### Advillin expression by sympathetics begins postnatally

**Figure 9.**
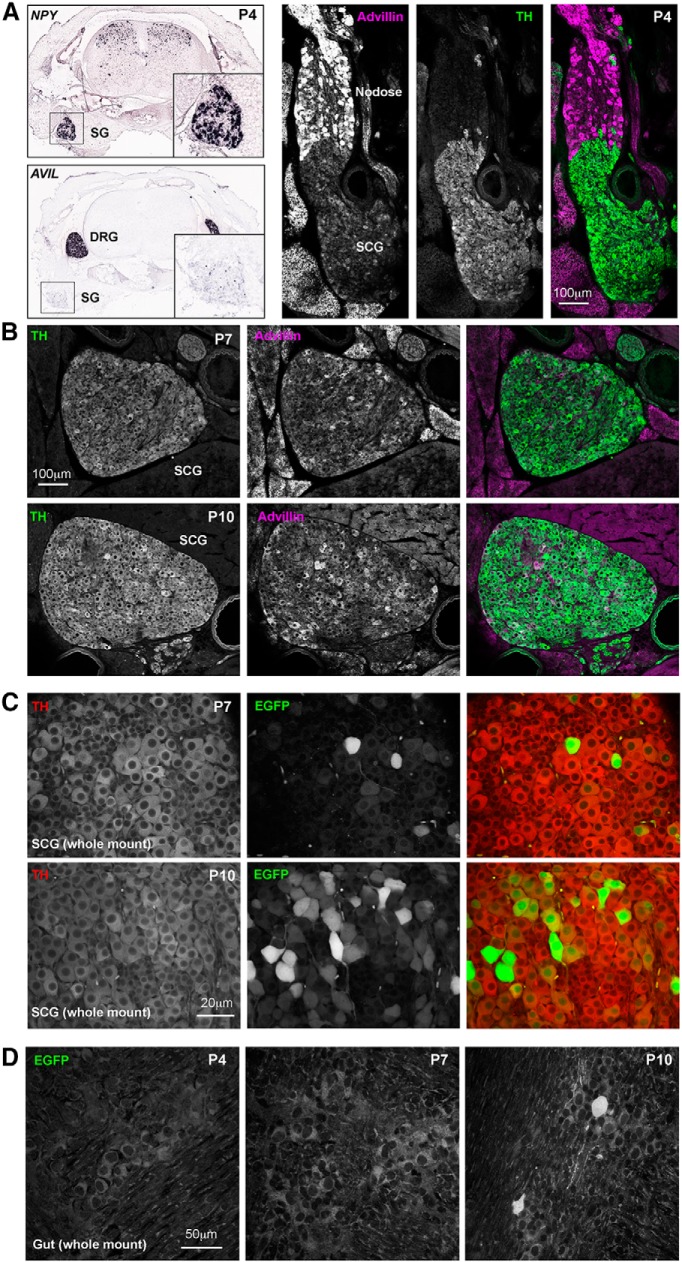
Advillin expression emerges postnatally in sympathetic neurons. ***A***, left, Allen Institute NPY ISH data showing the position of the sympathetic chain in lower cervical/upper thoracic spines; advillin mRNA signal intensity is low or absent in sympathetics at this time. ***A***, right, Advillin immunoreactivity is present in the nodose ganglion but absent from the fused sympathetic ganglion. ***B***, Advillin immunoreactivity in superior cervical ganglia of P7 and P10 mouse pups (30-μm sections). ***C***, Advillin-driven EGFP fluorescence also appears by day 7 and increases thereafter. ***D***, Advillin-driven EGFP expression is absent from enteric neurons before day 10, at which point advillin-positive neurons are still rare. Image credits in ***A***, left: Allen Institute (http://mousespinal.brain-map.org/imageseries/show.html?id=100040017; http://mousespinal.brain-map.org/imageseries/show.html?id=100020083).

Previous work by Hasegawa et al. (2007) showed convincingly a lack of advillin mRNA expression (or hPLAP staining) by sympathetic ganglion neurons in pre-natal mice (their Figure S2), and so we asked at what developmental point advillin begins to be expressed in this population. Fortuitously, Allen brain atlas *in situ* hybridization studies with P4 mice (alleninstitute.org) include sympathetic chain ganglia in their rostral sections of whole spines (Fig. 9*A*, left). These revealed a dearth of advillin mRNA in the P4 sympathetic chain, and so we examined mouse pups at P4, P7, and P10. In P4 mice, while advillin immunoreactivity was intense in nodose ganglia (containing primary afferents of the vagus nerve), it was absent from the adjoining superior cervical ganglion (SCG; Fig. 9*A*, right). Advillin immunoreactivity emerged in the SCG over the next 6 d (Fig. 9*B*). Similarly, few sympathetic neurons were EGFP-positive in P7 advillin-EGFP mice, but were abundant in P10 mice. In enteric neurons, advillin expression began later; absent at P7, and only occasional neurons positive at P10 (Fig. 9*D*).

## Discussion

Since the development of advillin-Cre mouse lines (Lau et al., 2011; Zurborg et al., 2011), the results of more than two dozen papers have relied on them for labeling or knocking out genes specifically in peripheral sensory neurons. In many cases, specificity of advillin expression to the DRG is important in their interpretation, but evidence thereof has been incomplete. We therefore conducted a thorough examination of the expression patterns of advillin in the adult mouse using immunohistochemistry and two advillin reporter mouse strains. Although advillin is expressed in all DRG neurons, we found that the expression was enriched in small, IB4-binding DRG neurons, corresponding to a subpopulation of non-peptidergic nociceptors (Snider and McMahon, 1998). Subsequent examination of autonomic neurons revealed substantial advillin expression in enteric, parasympathetic and sympathetic neurons as well as in their peripheral projections. The expression of advillin was particularly notable in fibers innervating blood vessels and in Merkel cells of glabrous and hairy skin. We extended these observations to the peripheral autonomic nervous system of rats and non-human primates. Finally, we show that advillin expression only emerges postnatally in the mouse autonomic nervous system.

Although advillin is ubiquitously expressed by DRG neurons, it has gone unnoticed that some early work indicated that it is not, in fact, sensory neuron-specific. The initial description of advillin expression in mice by Northern blot and *in situ* hybridization analyses revealed that in addition to the DRG and trigeminal ganglia, advillin is expressed in uterus, intestines, taste buds, and at low levels in the brain (Marks et al., 1998). Furthermore, expression data for pervin, the rat homolog of the advillin protein, showed expression extensively in both the DRG and SCG (Ravenall et al., 2002). The initial characterization of the advillin-Cre mouse also showed significant levels of Cre expression within the adult SCG (Zurborg et al., 2011). These early but clear indications of advillin expression in autonomic structures appear to have been subsequently overlooked. A reason might simply be anatomy: compared to easily-identifiable DRGs in the bony confines of the spinal column and the trigeminal ganglia in the base of the skull (Devor, 1999), the autonomic ganglia are widely-dispersed in the soft tissue of the animal and more challenging to isolate. For example, even the relatively large SCG can easily be confused by the inexperienced with nearby lymph nodes. The more likely explanation is that conclusions about advillin distribution have relied on prenatal expression patterns (Hasegawa et al., 2007; Minett et al., 2012). The present work not only underscores that advillin is expressed in adult autonomic neurons (Zurborg et al., 2011), but also identifies the first postnatal week as the time at which advillin expression by sympathetic neurons begins, and the second week for enteric neurons.

The lack of specificity of advillin expression is in some cases without significant issue. For example, the only spinal cord-projecting advillin-expressing axons are those of primary afferents which terminate throughout the gray matter and form the ascending dorsal columns. As such, studies that use advillin driven reporter expression to examine the termination patterns of sensory neurons in the spinal cord are able to take advantage of this mouse while avoiding potential pitfalls (Leslie et al., 2011; Comer et al., 2015; Abraira et al., 2017). Similarly, studies which use advillin-Cre expression tolabel neurons or knock-out genes *in vivo* before studying the effect within DRG neurons *in vitro*, circumvent the problems with additional gene expression changes in other cell types (Abe et al., 2010; Lopes et al., 2017). Combinatorial approaches taking advantage of expression of multiple genes have been used to effect changes in a more specific cell population than advillin alone. For example, McCoy et al. used an advillin-Cre mouse crossed with a LoxP-stopped human diphtheria receptor knocked-in to the CGRPα locus so that CGRP-expressing sensory neurons were specifically targeted for ablation with diphtheria toxin (McCoy et al., 2013; McCoy and Zylka, 2014). Requiring the expression of multiple genes for recombination may be the key to specifically targeting the entire population of DRG neurons.

Caution, however, must be applied when interpreting results where multiple advillin-positive populations are involved, such as *in vivo* regeneration studies, or where changes in behavioral responses or target tissue innervation are examined (da Silva et al., 2011; Lee et al., 2014; Minett et al., 2014; Weng et al., 2015). In some cases, this may mean simply extending conclusions to include additional cell populations. For example, Shin et al. (2014) used advillin-driven reporter expression in axons to conclude that SCG10 is present at high levels in sensory, but not motor axons; our results (not to mention the fact that SCG10 stands for superior cervical ganglion 10), allow inclusion of sympathetics among SCG10-enriched axons. However, for cases where the independent roles of sensory and autonomic neurons need to be considered separately, such as in some studies of pain and neuroinflammation, the expression of advillin throughout the peripheral nervous system could significantly impact the conclusions. In one such study (Minett et al., 2012), the role of (floxed) Nav1.7 in different pain modalities was examined using mouse lines that had Cre expression driven by either Wnt1, which is expressed in all neural crest derived cells, Nav1.8, which is expressed in a subset of sensory neurons, or advillin. Interestingly, only the Wnt1-Cre mice demonstrated a “pain-free” phenotype, which was inferred as due to the elimination of Nav1.7 in both sensory and sympathetic neurons. However, based on the fact that advillin is expressed in peripheral sympathetic neurons, an alternate explanation for their findings must be found. Feng et al., used advillin-driven channelrhodopsin 2 to “selectively” stimulate sensory axons projecting to the colon *via* the pelvic nerve (Feng et al., 2016). Not only does the pelvic nerve convey sympathetic and parasympathetic input to the colon from the pelvic ganglion, but it also contains centrifugally-projecting axons of enteric neurons (Luckensmeyer and Keast, 1996), all of which would be expected in this case to be activated by light. It is clear from these examples that the key to drawing conclusions from *in vivo* studies that use genetically modified mice is in understanding the complete impact of the modifications. Indeed, a recent study took advantage of advillin-Cre expression in both sensory neurons and Merkel cells, to demonstrate the importance of Piezo_2_ expression in transduction of low-threshold mechanical stimuli (Ranade et al., 2014).

Although the expression of advillin in multiple cells types limits its use as a way to specifically target primary sensory neurons, modification of techniques may help to restrict gene manipulation to desired populations. Attempts to only activate recombination in primary sensory neurons might be undertaken in advillin-CreERT2 mice via intrathecal administration of tamoxifen methiodide (which unlike tamoxifen, does not easily cross the blood-brain-barrier (Biegon et al., 1996; Sömjen et al., 1996). A second method might be the intrathecal administration of specific serotypes of recombinant adeno-associated virus (Storek et al., 2006; Mason et al., 2010; Vulchanova et al., 2010; Machida et al., 2013; Fagoe et al., 2014) carrying advillin promoter-driven Cre, which would result in Cre expression only in the infected cells that are also expressing advillin. However, difficulties with achieving high-level, long last lasting gene expression within all DRGs by intrathecal injection may mean lower recombination efficiency in the peripheral sensory neuron population (Beutler et al., 2005; Vulchanova et al., 2010). An alternative is suggested by our developmental data: conditional recombination using advillin-CreERT2 mice before postnatal day 4 would be expected to spare autonomic neurons.

In conclusion, our data clearly show that in mammals, advillin is expressed in the all classes of neural crest-derived neurons; advillin-driven expression of reporters or recombinases is not sensory neuron specific. Genetic labeling or modification based on advillin expression remains useful, but must take in to account or exclude potentially important roles of other advillin-expressing cells.

## Synthesis

Reviewing Editor: Mike Fainzilber, Weizmann Institute of Science Decisions are customarily a result of the Reviewing Editor and the peer reviewers coming together and discussing their recommendations until a consensus is reached. When revisions are invited, a fact-based synthesis statement explaining their decision and outlining what is needed to prepare a revision will be listed below. The following reviewer(s) agreed to reveal their identity: Paul Heppenstall. Note: If this manuscript was transferred from JNeurosci and a decision was made to accept the manuscript without peer review, a brief statement to this effect will instead be what is listed below.

In this manuscript, the expression of the actin-binding protein advillin is examined across several neuronal and non-neuronal tissues in mice, rats and non-human primates. The authors find that advillin is expressed fairly broadly and can be found in most neural-crest derived cells. This finding is important because the advillin locus is widely used to drive Cre recombinase expression for sensory neuron specific recombination. The paper points out that advillin promoter driven Cre expression will also lead to recombination in other cell types including the autonomic and enteric nervous systems, the habenula and various endocrine cells of the gut and skin. While this finding is not particularly novel (several other groups have described advillin expression beyond the peripheral nervous system), it is timely in that it has become almost dogma that advillin Cre driver lines are sensory neuron specific. However, there are some concerns that must be addressed before a final decision regarding publication, as follows:

1) Validation of the advillin antibody must be done on advillin knockout tissues as controls. This is critical given the main message of the paper, as otherwise doubts may still remain.

2) The paper should be strengthened by inclusion of developmental data. This can be done by immunostaining of embryos at E15-16 or by labeling using the Tg(Avil-icre/ERT2). In Hasegawa et.al, it is clearly demonstrated that Advillin is not expressed in the SCG (by in situ and KI PLAP reporter). It might well be that there is a window in which the expression is sensory neurons specific.

3) The authors tend to overstate the novelty of their work and are too strong in their condemnation of previous work which reported sensory-neuron specific expression of advillin, for example on page 5 and 6. Similarly, Zurborg et al found expression of advillin in other tissues contrary to what might be assumed from paragraph 2 on page 5, and this should be made more explicit. Citations of the original advillin-Cre mouse (Hasegawa et al) are also sometimes missing (e.g. first line of the discussion). By toning down assertions about previous literature, and perhaps discussing technical differences which might arise from using different mouse lines (knock-ins versus BAC transgenics) and different protocols for tamoxifen induction, the message that advillin is expressed beyond DRG neurons would still be clear.

4) The authors sometimes include data from public resources in their figures (Figure 1 and Figure 3). It would be more convincing if original data could be included and this data be removed. This is most apparent in Figure 3. Rather than the inset in 3A, it would be quite simple to include an original image at the same spinal level from the advillin-GFP mouse line used in this study. Similarly, the table in 3D does not add much information, and if the authors wish to explore Avil expression in different populations of DRG neurons then immunohistochemistry experiments should be performed with the appropriate marker antibodies.

5) The authors should provide more information about the Tg animals they used. Are these transgenic, BAC-transgenic or a knockin?

6) The authors do not refer to Fig. 5 in the text?

## Author Response

Dear Dr. Fainzilber,

Thank you very much for the opportunity to submit a revised version of our manuscript. The MS has been revised according to the reviewers' suggestions. We have also performed an additional developmental study as suggested by the reviewer(s), which we feel has greatly improved the mansuscript.

Comment:

1) Validation of the advillin antibody must be done on advillin knockout tissues as controls. This is critical given the main message of the paper, as otherwise doubts may still remain.

Response:

This would certainly be true were ISH data, other antibodies, and reporter mice not available. The developmental data we now include provide further support. First, the antibody data match precisely with ISH data in brain and DRG (Allen institute ISH data shown in Fig. 1). Second, advillin immunohistochemistry mirrors Advillin-EGFP signal not only in terms of tissue distribution, but also in terms of intensity in the DRG (note tight correlation in Fig. 1), and temporal expression (Figure 9, where we show postnatal onset of advillin expression in sympathetics). Third, mouse duodenal staining with the antibody we used (abcam ab72210) matches immunohistochemical staining in the human duodenum using a diffierent advillin antibody (Atlas antibodies HPA058864). Fourth, both reporter lines, which are different BAC transgenics, show overlap in the appropriate tissues. It might be argued that in either strain (advillin-EGFP and advillin-CreERT2), the site of integration and copy number of the BAC transgenes are unknown, which might lead to a degree of uncertainty as to whether local chromatin environment, nearby genes or extra copies of advillin might distort native expression patterns. However, both variables are also extremely likely to differ between the two lines, since construct integration and concatemerization are determined by chance during the generation of founder mice. Any overlap in expression (of EGFP or CreERT2) is therefore likely to reflect “real advillin signal” and independent of possible extraneous artefacts specific to a particular transgenic strain. Together, these data render any concern about the specificity of the antibody vanishingly small. This has been clarified in the text.

Comment:

2) The paper should be strengthened by inclusion of developmental data. This can be done by immunostaining of embryos at E15-16 or by labeling using the Tg(Avil-icre/ERT2). In Hasegawa et.al, it is clearly demonstrated that Advillin is not expressed in the SCG (by in situ and KI PLAP reporter). It might well be that there is a window in which the expression is sensory neurons specific.

Response:

This has turned out to be an excellent suggestion. We went back to Allen Institute ISH data, and found that in their P4 cord samples (whole spine), the upper thoracic sympathetic chain was often included. At P4, advillin mRNA is expressed at very low levels in the sympathetic chain (Fig. 9A). We therefore looked at pups aged 4 days, 7 days and 10 days, and show that both advillin immunoreactivity and advillin-driven EGFP fluorescence emerge during the post-natal period. We have changed the title of the paper to reflect that advillin is not sensory neuron specific in the adult mouse. This has also led to solutions for genetic manipulation using advillin-CreERT2: i.e. treat with tamoxifen prior to P4.

Comment:

3) The authors tend to overstate the novelty of their work and are too strong in their condemnation of previous work which reported sensory-neuron specific expression of advillin, for example on page 5 and 6. Similarly, Zurborg et al found expression of advillin in other tissues contrary to what might be assumed from paragraph 2 on page 5, and this should be made more explicit. Citations of the original advillin-Cre mouse (Hasegawa et al) are also sometimes missing (e.g. first line of the discussion). By toning down assertions about previous literature, and perhaps discussing technical differences which might arise from using different mouse lines (knock-ins versus BAC transgenics) and different protocols for tamoxifen induction, the message that advillin is expressed beyond DRG neurons would still be clear.

Response:

We have toned-down our criticisms of previous literature, although it is important to emphasize that the dogmatic notion of advillin as sensory specific is mistaken, which has led to some conclusions that need revisiting. The new developmental experiments add novelty, and point not to differences in BAC transgenic lines, but developmental differences between DRGs, sympathetics, and enteric neurons in terms of advillin expression.

The Hasegawa paper did not use an advillin-cre mouse. Rather they generated an advillin-hPLAP mouse.

Comment:

4) The authors sometimes include data from public resources in their figures (Figure 1 and Figure 3). It would be more convincing if original data could be included and this data be removed. This is most apparent in Figure 3. Rather than the inset in 3A, it would be quite simple to include an original image at the same spinal level from the advillin-GFP mouse line used in this study. Similarly, the table in 3D does not add much information, and if the authors wish to explore Avil expression in different populations of DRG neurons then immunohistochemistry experiments should be performed with the appropriate marker antibodies.

Response:

The purpose of publicly available datasets is to avoid re-inventing the wheel. However, we have now included “original” advillin-EGFP data in Figs 1 and 3. Not surprisingly, these match the publicly available data, since they are the same transgenic line. We have kept the parasagittal section from the Gensat database as it nicely demonstrates the habenula and fasciculus retroflexus. We have also kept the Allen ISH data for the habenula as it strengthens the argument that the antibody is specific.

We have definitively shown that the DRG neuronal subpopulation that expresses advillin at high levels is the non-peptidergic (IB4-binding) phenotype. The evidence for this is (1) the pattern of advillin staining in the dorsal horn of the spinal cord in lamina IIi, and (2) the tight correlation between IB4-binding intensity and advillin intensity (both Figure 3). It is well-established that the IB4-population has little overlap with the other neuronal phenotypes in the mouse (peptidergic nociceptors, NF200 cells, TH-positive neurons), and so repeating this experiment with markers for those populations would be redundant. Although the Usoskin-Ernfors table provides further information about the supopulation of non-peptidergic cells with high advillin expression, we have removed it for clarity.

Comment:

5) The authors should provide more information about the Tg animals they used. Are these transgenic, BAC-transgenic or a knockin?

Response:

Both the advillin-EGFP and Advillin-CreERT2 lines are BAC transgenics. This has been clarified.

Comment:

6) The authors do not refer to Fig. 5 in the text?

Response:

We do indeed refer to Fig. 5 (highlighted text in the revised version).

## References

[B1] Abe N, Borson SH, Gambello MJ, Wang F, Cavalli V (2010) Mammalian target of rapamycin (mTOR) activation increases axonal growth capacity of injured peripheral nerves. J Biol Chem 285:28034–28043. 10.1074/jbc.M110.125336 20615870 PMC2934668

[B2] Abraira VE, Kuehn ED, Chirila AM, Springel MW, Toliver AA, Zimmerman AL, Orefice LL, Boyle KA, Bai L, Song BJ, Bashista KA, O'Neill TG, Zhuo J, Tsan C, Hoynoski J, Rutlin M, Kus L, Niederkofler V, Watanabe M, Dymecki SM, et al. (2017) The cellular and synaptic architecture of the mechanosensory dorsal horn. Cell 168:295–310.e19. 10.1016/j.cell.2016.12.010 28041852 PMC5236062

[B3] Al-Hadithi BA, Mitchell J (1987) The otic ganglion and its neural connections in the rat. J Anat 154:113–119. 3446656 PMC1261841

[B4] Beutler AS, Banck MS, Walsh CE, Milligan ED (2005) Intrathecal gene transfer by adeno-associated virus for pain. Curr Opin Mol Ther 7:431–439. 16248278

[B5] Biegon A, Brewster M, Degani H, Pop E, Somjen D, Kaye AM (1996) A permanently charged tamoxifen derivative displays anticancer activity and improved tissue selectivity in rodents. Cancer Res 56:4328–4331. 8813117

[B6] Chiu IM, Barrett LB, Williams EK, Strochlic DE, Lee S, Weyer AD, Lou S, Bryman GS, Roberson DP, Ghasemlou N, Piccoli C, Ahat E, Wang V, Cobos EJ, Stucky CL, Ma Q, Liberles SD, Woolf CJ (2014) Transcriptional profiling at whole population and single cell levels reveals somatosensory neuron molecular diversity. Elife 3:04660. 10.7554/eLife.06720 PMC4383053 25525749

[B7] Comer JD, Pan FC, Willet SG, Haldipur P, Millen KJ, Wright CV, Kaltschmidt JA (2015) Sensory and spinal inhibitory dorsal midline crossing is independent of Robo3. Front Neural Circuits 9:36. 10.3389/fncir.2015.00036 26257608 PMC4511845

[B8] da Silva S, Hasegawa H, Scott A, Zhou X, Wagner AK, Han BX, Wang F (2011) Proper formation of whisker barrelettes requires periphery-derived Smad4-dependent TGF-beta signaling. Proc Natl Acad Sci USA 108:3395–3400. 10.1073/pnas.1014411108 21300867 PMC3044401

[B9] Devor M (1999) Unexplained peculiarities of the dorsal root ganglion. Pain 6:S27–S35. 10491970 10.1016/S0304-3959(99)00135-9

[B10] Edgar MA (2007) The nerve supply of the lumbar intervertebral disc. J Bone Joint Surg Br 89:1135–1139. 10.1302/0301-620X.89B9.18939 17905946

[B11] Fagoe ND, Eggers R, Verhaagen J, Mason MR (2014) A compact dual promoter adeno-associated viral vector for efficient delivery of two genes to dorsal root ganglion neurons. Gene Ther 21:242–252. 10.1038/gt.2013.71 24285216

[B12] Feng B, Joyce SC, Gebhart GF (2016) Optogenetic activation of mechanically insensitive afferents in mouse colorectum reveals chemosensitivity. Am J Physiol Gastrointest Liver Physiol 310:G790–G798. 10.1152/ajpgi.00430.2015 26950857 PMC4888546

[B13] Hasegawa H, Abbott S, Han BX, Qi Y, Wang F (2007) Analyzing somatosensory axon projections with the sensory neuron-specific advillin gene. J Neurosci 27:14404–14414. 10.1523/JNEUROSCI.4908-07.2007 18160648 PMC6673440

[B14] Lau J, Minett MS, Zhao J, Dennehy U, Wang F, Wood JN, Bogdanov YD (2011) Temporal control of gene deletion in sensory ganglia using a tamoxifen-inducible advillin-Cre-ERT2 recombinase mouse. Mol Pain 7:100. 10.1186/1744-8069-7-100 22188729 PMC3260248

[B15] Lee B, Cho H, Jung J, Yang YD, Yang DJ, Oh U (2014) Anoctamin 1 contributes to inflammatory and nerve-injury induced hypersensitivity. Mol Pain 10:5. 10.1186/1744-8069-10-5 24450308 PMC3929161

[B16] Leslie JR, Imai F, Fukuhara K, Takegahara N, Rizvi TA, Friedel RH, Wang F, Kumanogoh A, Yoshida Y (2011) Ectopic myelinating oligodendrocytes in the dorsal spinal cord as a consequence of altered semaphorin 6D signaling inhibit synapse formation. Development 138:4085–4095. 10.1242/dev.066076 21831918 PMC3160102

[B17] Lopes DM, Denk F, McMahon SB (2017) The molecular fingerprint of dorsal root and trigeminal ganglion neurons. Front Mol Neurosci 10:304. 10.3389/fnmol.2017.00304 29018326 PMC5623188

[B18] Luckensmeyer GB, Keast JR (1996) Immunohistochemical characterisation of viscerofugal neurons projecting to the inferior mesenteric and major pelvic ganglia in the male rat. J Auton Nerv Syst 61:6–16. 10.1016/0165-1838(96)00056-2 8912248

[B19] Machida A, Kuwahara H, Mayra A, Kubodera T, Hirai T, Sunaga F, Tajiri M, Hirai Y, Shimada T, Mizusawa H, Yokota T (2013) Intraperitoneal administration of AAV9-shRNA inhibits target gene expression in the dorsal root ganglia of neonatal mice. Mol Pain 9:36. 10.1186/1744-8069-9-36 23866078 PMC3737086

[B20] Marks PW, Arai M, Bandura JL, Kwiatkowski DJ (1998) Advillin (p92): a new member of the gelsolin/villin family of actin regulatory proteins. J Cell Sci 111:2129–2136. 9664034 10.1242/jcs.111.15.2129

[B21] Mason MR, Ehlert EM, Eggers R, Pool CW, Hermening S, Huseinovic A, Timmermans E, Blits B, Verhaagen J (2010) Comparison of AAV serotypes for gene delivery to dorsal root ganglion neurons. Mol Ther 18:715–724. 10.1038/mt.2010.19 20179682 PMC2862541

[B22] McCoy ES, Zylka MJ (2014) Enhanced behavioral responses to cold stimuli following CGRPα sensory neuron ablation are dependent on TRPM8. Mol Pain 10:69. 10.1186/1744-8069-10-69 25406633 PMC4247560

[B23] McCoy ES, Taylor-Blake B, Street SE, Pribisko AL, Zheng J, Zylka MJ (2013) Peptidergic CGRPα primary sensory neurons encode heat and itch and tonically suppress sensitivity to cold. Neuron 78:138–151. 10.1016/j.neuron.2013.01.030 23523592 PMC3628403

[B24] Minett MS, Nassar MA, Clark AK, Passmore G, Dickenson AH, Wang F, Malcangio M, Wood JN (2012) Distinct Nav1.7-dependent pain sensations require different sets of sensory and sympathetic neurons. Nat Commun 3:791. 10.1038/ncomms1795 22531176 PMC3337979

[B25] Minett MS, Falk S, Santana-Varela S, Bogdanov YD, Nassar MA, Heegaard AM, Wood JN (2014) Pain without nociceptors? Nav1.7-independent pain mechanisms. Cell Rep 6:301–312. 10.1016/j.celrep.2013.12.033 24440715 PMC3969273

[B26] Niu J, Ding L, Li JJ, Kim H, Liu J, Li H, Moberly A, Badea TC, Duncan ID, Son Y-J, Scherer SS, Luo W (2013) Modality-based organization of ascending somatosensory axons in the direct dorsal column pathway. J Neurosci 33:17691–17709. 10.1523/JNEUROSCI.3429-13.2013 24198362 PMC3818546

[B27] Pongratz G, Straub RH (2014) The sympathetic nervous response in inflammation. Arthritis Res Ther 16:504. 25789375 10.1186/s13075-014-0504-2 PMC4396833

[B28] Raja SN (1995) Role of the sympathetic nervous system in acute pain and inflammation. Ann Med 27:241–246. 7632421 10.3109/07853899509031966

[B29] Ranade SS, Woo SH, Dubin AE, Moshourab RA, Wetzel C, Petrus M, Mathur J, Bégay V, Coste B, Mainquist J, Wilson AJ, Francisco AG, Reddy K, Qiu Z, Wood JN, Lewin GR, Patapoutian A (2014) Piezo2 is the major transducer of mechanical forces for touch sensation in mice. Nature 516:121–125. 10.1038/nature13980 25471886 PMC4380172

[B30] Ravenall SJ, Gavazzi I, Wood JN, Akopian AN (2002) A peripheral nervous system actin-binding protein regulates neurite outgrowth. Eur J Neurosci 15:281–290. 11849295 10.1046/j.0953-816x.2001.01862.x

[B31] Shin JE, Geisler S, DiAntonio A (2014) Dynamic regulation of SCG10 in regenerating axons after injury. Exp Neurol 252:1–11. 10.1016/j.expneurol.2013.11.007 24246279 PMC3947015

[B32] Silacci P, Mazzolai L, Gauci C, Stergiopulos N, Yin HL, Hayoz D (2004) Gelsolin superfamily proteins: key regulators of cellular functions. Cell Mol Life Sci 61:2614–2623. 10.1007/s00018-004-4225-6 15526166 PMC11924436

[B33] Snider WD, McMahon SB (1998) Tackling pain at the source: new ideas about nociceptors. Neuron 20:629–632. 9581756 10.1016/s0896-6273(00)81003-x

[B34] Sömjen D, Waisman A, Kaye AM (1996) Tissue selective action of tamoxifen methiodide, raloxifene and tamoxifen on creatine kinase B activity in vitro and in vivo. J Steroid Biochem Mol Biol 59:389–396. 9010344 10.1016/s0960-0760(96)00135-5

[B35] Stirling LC, Forlani G, Baker MD, Wood JN, Matthews EA, Dickenson AH, Nassar MA (2005) Nociceptor-specific gene deletion using heterozygous NaV1.8-Cre recombinase mice. Pain 113:27–36. 10.1016/j.pain.2004.08.015 15621361

[B36] Storek B, Harder NM, Banck MS, Wang C, McCarty DM, Janssen WG, Morrison JH, Walsh CE, Beutler AS (2006) Intrathecal long-term gene expression by self-complementary adeno-associated virus type 1 suitable for chronic pain studies in rats. Mol Pain 2:4. 10.1186/1744-8069-2-4 16445862 PMC1373607

[B37] Usoskin D, Furlan A, Islam S, Abdo H, Lönnerberg P, Lou D, Hjerling-Leffler J, Haeggström J, Kharchenko O, Kharchenko PV, Linnarsson S, Ernfors P (2015) Unbiased classification of sensory neuron types by large-scale single-cell RNA sequencing. Nat Neurosci 18:145–153. 10.1038/nn.3881 25420068

[B38] Vulchanova L, Schuster DJ, Belur LR, Riedl MS, Podetz-Pedersen KM, Kitto KF, Wilcox GL, McIvor RS, Fairbanks CA (2010) Differential adeno-associated virus mediated gene transfer to sensory neurons following intrathecal delivery by direct lumbar puncture. Mol Pain 6:31. 10.1186/1744-8069-6-31 20509925 PMC2900238

[B39] Wang L, Mongera A, Bonanomi D, Cyganek L, Pfaff SL, Nüsslein-Volhard C, Marquardt T (2014) A conserved axon type hierarchy governing peripheral nerve assembly. Development 141:1875–1883. 10.1242/dev.106211 24700820 PMC13148187

[B40] Weng HJ, Patel KN, Jeske NA, Bierbower SM, Zou W, Tiwari V, Zheng Q, Tang Z, Mo GC, Wang Y, Geng Y, Zhang J, Guan Y, Akopian AN, Dong X (2015) Tmem100 is a regulator of TRPA1-TRPV1 complex and contributes to persistent pain. Neuron 85:833–846. 10.1016/j.neuron.2014.12.065 25640077 PMC4336228

[B41] Zurborg S, Piszczek A, Martínez C, Hublitz P, Al Banchaabouchi M, Moreira P, Perlas E, Heppenstall PA (2011) Generation and characterization of an Advillin-Cre driver mouse line. Mol Pain 7:66. 10.1186/1744-8069-7-66 21906401 PMC3185264

